# Maxillary Sinus Floor Augmentation Using an Equine-Derived Graft Material: Preliminary Results in 17 Patients

**DOI:** 10.1155/2017/9164156

**Published:** 2017-10-25

**Authors:** F. Rivara, M. Negri, S. Lumetti, L. Parisi, A. Toffoli, E. Calciolari, E. Manfredi, G. M. Macaluso

**Affiliations:** ^1^Dipartimento di Medicina e Chirurgia, Centro di Odontoiatria, Università di Parma, Parma, Italy; ^2^Private Practice, Piacenza, Italy; ^3^Centre for Oral Clinical Research and Centre for Oral Immunobiology and Regenerative Medicine, Institute of Dentistry, Barts and The London School of Medicine and Dentistry, QMUL, London, UK

## Abstract

**Objective:**

Sinus floor elevation with lateral approach is probably the most frequently performed reconstructive procedure to rehabilitate posterior maxilla when a bone deficiency is present. Different graft materials have been proposed and tested, often with high clinical performances and predictable results. Histological analysis is required when evaluating new materials. We investigated human biopsies retrieved after sinus floor elevation procedure by histomorphometric evaluation to test the performance of an equine-derived bone grafting material.

**Study Design:**

Seventeen consecutive patients were enrolled and sinus lift surgeries were performed using an equine bone graft. Six months after surgery, at implant placement, bone samples were collected. Histomorphometry analysis was carried out on decalcified samples.

**Results:**

All surgeries were uneventful and no additional grafting was required prior to implant insertion. Forty percent of new bone formation was detected, which represented the most abundant tissue retrieved, followed by the residual graft material (33%) and fibrous tissue (27%). A significant reduction in particles size demonstrates a remodeling activity of the graft material.

**Conclusion:**

Within the limitations of this study, this equine-derived bone graft proved to be an effective material to induce new bone formation in the sinus floor elevation procedure.

## 1. Introduction

Dental implants can be successfully inserted in patient affected by systemic disease as well as in deficient bone conditions [1]. In some challenging situations bone augmentation is the only option that allows the replacement of missing teeth with dental implants.

Sinus floor elevation, first introduced in 1976, then revised between 1980 and 1986, has become one of the most common methods to increase bone height in deficient posterior alveolar ridges [2–4]. In particular, maxillary sinus floor elevation using a lateral approach is the most frequently used surgical technique to increase bone volume in the posterior area. Previous studies showed that implants associated with sinus lift have a predictable long-term success and survival [5]. A systematic review of the literature showed that graftless sinus elevation may ensure predictable bone formation [6]. However, the use of autogenous bone grafts in sinus augmentation is considered the best choice to obtain bone regeneration [7, 8]. It has shown an excellent survival rate for loaded implants. Nevertheless, drawbacks of using autologous bone grafts include patient's morbidity, risk of infection in the donor site, pain, and blood loss [9]. As an alternative to autologous bone grafts, surgeons can choose between three categories of bone substitutes: xenogenic grafts (derived from other species), allogenic grafts (from the same species), and alloplastic materials (synthetic materials). They all share the great advantage of unlimited availability and avoidance of invasive harvesting procedures required for autologous bone [8–10]. Deproteinized bovine bone mineral (DBBM), introduced in 1995 for sinus lift, is the most commonly used xenogenic graft, either alone or mixed with autogenous bone or Platelet Rich Plasma (PRP) [11].

Amongst allogenic grafts derived from same-species donors, Demineralized Freeze-Dried Bone (DFDB) and Fresh-Frozen Bone (FFB) are two of the best-documented graft materials for sinus lift or for ridge augmentation [12–14]. Alloplastic grafts are synthetic biocompatible materials and include, for example, hydroxyapatite (HA), proposed in 1987, tricalcium phosphate (TCP) proposed in 1986, or bioactive glasses [4, 15, 16].

According to the available evidence, the survival rate of implants placed in association with sinus elevation (one-stage surgery) does not significantly differ when autogenous or nonautogenous or mixed bone grafts are used [17, 18].

Recently, equine-derived cancellous bone substitute was introduced as a scaffold for bone regeneration. A preclinical study employing this type of graft for alveolar ridge reconstruction showed a weak new bone formation and a massive connective tissue invasion [19].

The aim of this prospective study was to perform a histological and histomorphometric evaluation of an equine-derived xenogenic material employed for lateral approach sinus floor augmentation at the time of implant insertion, that is, 6 months after the grafting procedure.

## 2. Materials and Methods

### 2.1. Patient Selection

Seventeen patients referred to a Private Practice specialized in implant dentistry and bone reconstruction for rehabilitation of a partially or totally edentulous maxilla with an implant-supported fixed prosthesis and requiring sinus augmentation surgery were consecutively enrolled for this study. All patients met the following inclusion criteria:

1. Good general physical and mental health
2. Nonsmoker
3. Good systemic health
4. No active periodontitis
5. Residual alveolar bone height ≤ 3 mm
6. Need for two-stage sinus augmentation
7. Age > 18 years
8. No compromised general health (ASA I or II, American Society of Anesthesiology)
9. No drug/alcohol abuse
10. Sufficient prosthetic space
11. High level of oral hygiene (PI < 10%)
12. No systemic diseases severely affecting bone metabolism (e.g., Cushing's syndrome, Addison's disease, diabetes mellitus, leukemia, pernicious anemia, malabsorption syndromes, chronic liver disease, and rheumatoid arthritis).

All surgeries (sinus floor augmentation, implant placement, and biopsy retrieval) were performed by one experienced surgeon.

### 2.2. Surgery

One hour before surgery, patients started antibiotic therapy with 2 g amoxicillin clavulanate tablets (Augmentin, GlaxoSmithKline, Brentford, UK) and continued therapy with 1 g every 12 hours for 7 days after surgery. Prior to anesthesia, mouth was rinsed with chlorhexidine 0.2% (Corsodyl, GlaxoSmithKline, Brentford, UK) for one minute. Infiltrative anesthesia was performed using 4% mepivacaine with 1 : 100.000 epinephrine (Pierrel, Capua, CE, Italy).

After a crestal incision with mesial release, a full-thickness mucoperiosteal flap was raised to expose the lateral wall of the maxillary sinus. By using a fine diamond bur and under copious saline irrigation, osteotomy of the cortical bone was performed, and a lateral window was carefully prepared 2-3 mm above the margin between the junction of the alveolar process and the facial lateral maxillary sinus wall. The lateral wall and the Schneiderian membrane were then carefully raised avoiding injuries and lacerations to expose the bone surface using dedicated atraumatic instruments (Hu-Friedy, Chicago, IL, USA). The space created between the maxillary alveolar process and the elevated sinus floor was filled with an equine particulate graft (Bio-Gen Mix, Bioteck, Vercelli, Italy). Xenogenic bone cortical granules (0,5–1 mm) were soaked with sterile saline before grafting. A collagen membrane (Biocollagen, Bioteck, Vercelli, Italy) was placed on the vestibular osteotomy. Primary closure of the surgical wound was achieved by using a 4.0 PTFE suture (Omnia SPA, Fidenza, PR, Italy), and postsurgery antibiotics, analgesics, antibacterial mouthwash, and antihistaminic decongestants were prescribed. Sutures were removed after 7 to 10 days. All patients were recalled for weekly follow-up visits during the first month after surgery to assess the wound healing and for monthly recall visit during the following 6 months.

### 2.3. Implant Surgery and Biopsy

Six months after the sinus lift surgery, bioptic samples were harvested (one sample was analyzed for each patient) and implants were placed under local anesthesia. A horizontal incision at the top of the alveolar crest was followed by vertical incisions for tissue release. A full-thickness flap was raised and mobilised for tension-free closure. Bone was inspected, and biopsies (7–11 mm in depth) were harvested with a 2.5 mm diameter trephine burr (Thomas, Bourges, France) at the grafted sites, under irrigation with sterile saline. The biopsies were processed for decalcified histology. After biopsy collection, 3.8 or 4.25 mm diameter dental implants (Premium, Sweden & Martina, Due Carrare, PD, Italy) were placed into the implant beds and primary stability was achieved for all patients. The flap was sutured with 4-0 Vicryl polyglactin sutures (Ethicon, Somerville, NJ, USA). Sutures were removed after 7 to 10 days, and provisional prosthesis was connected. The implants were loaded with final porcelain restoration after a 6-month healing period.

### 2.4. Histologic Analysis

Biopsies were fixed in 10% formalin (pH 7) for 24 hours. The specimens were decalcified for 2 hours in Histo DECAL solution (Histoline) according to the manufacturer's instructions. After decalcification, specimens were dehydrated in a graded ethanol series and xylene, paraffin-embedded, and blocks were sliced into 3-4 *μ*m thick sections and adhered to poly-l-lysine-coated glass microscope slides. Samples were then stained with Hematoxyline-Eosin and Masson Trichrome staining. For histomorphometric analysis, the amount of bone, fibrous tissue, and residual material was quantified by a trained calibrated examiner. Additionally, we elaborated these results to calculate the ratios of bone volume/tissue volume (BV/TV) and fibrous tissue volume/tissue volume (FTV/TV).

### 2.5. Statistical Analysis

Due to the nonparametric distribution of the data, Kruskall-Wallis test was used to evaluate the statistical significance of the differences between tissue components, and results were considered significant when *p* < 0.05. Data were reported as mean ± standard deviation.

## 3. Results

All patients healed uneventfully, no infection occurred during healing, and no additional grafting was required prior to implant insertion.

**Figure 1:**
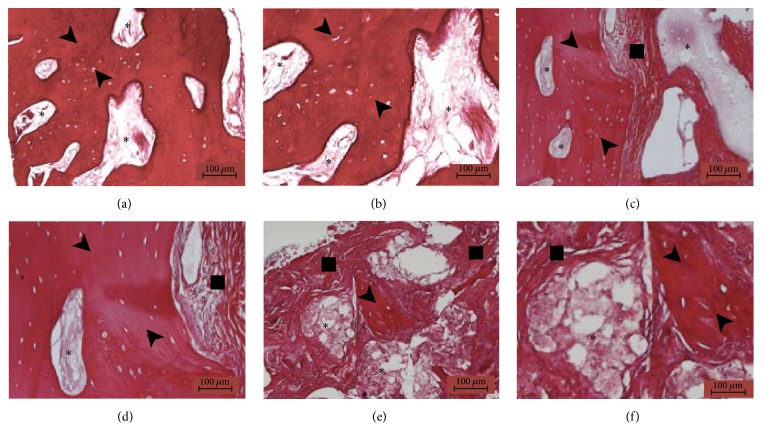
Histologic analysis: Hematoxylin-Eosin staining, magnification of 10x (a, c, e) and 20x (b, d, f). The histologic analysis reveals the presence of predominant bone tissue (black arrow), fibrous tissue (black square), and residual grafting material (black star). The grafting material is in direct contact with the bone that completely surrounds it (a, b) or partially engulfed in the newly formed fibrous tissue (c, d) or fragmented in small residual granules surrounded by a mixture of fibrous tissue and bone spicules (e, f).

**Figure 2:**
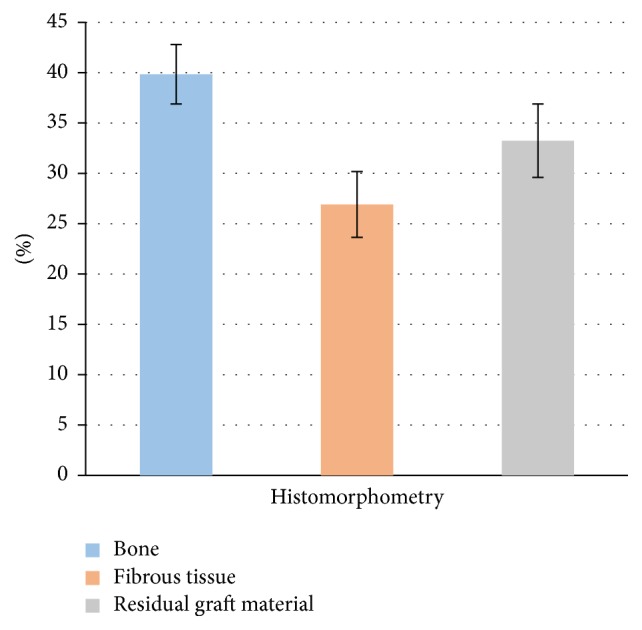
Histomorphometric analysis: the chart represents the percentage with standard deviation of bone, fibrous tissue, and residual graft material in the biopsy sample at 6 months.

**Figure 3:**
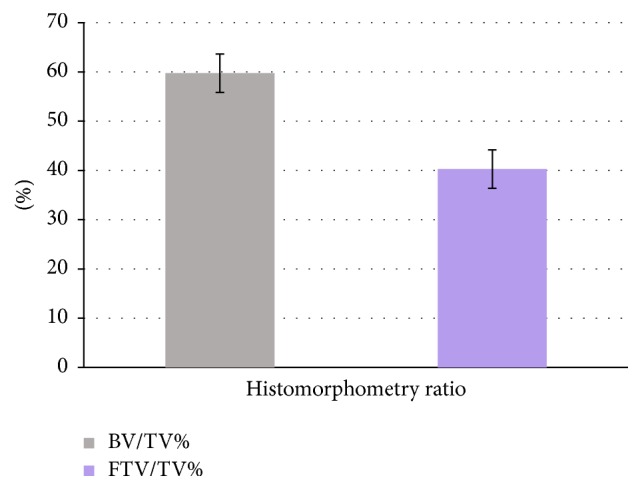
Histomorphometric analysis: the diagram shows the ratio between bone volume (BV) and total volume of the sample (TV, grey) and the ratio between fibrous tissue volume (FTV) and total volume of the sample (TV, violet).

**Figure 4:**
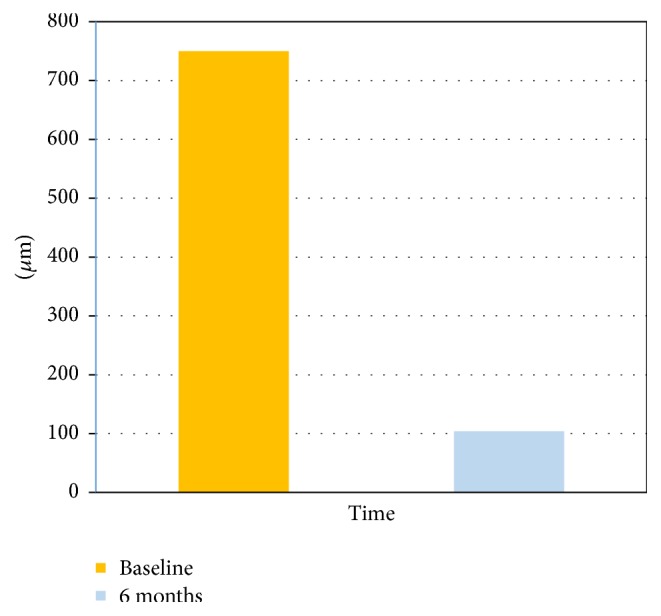
Granules size analysis: grafting material granules' size at six months compared to the baseline (data provided by the manufacturer).

Histological analysis (Figure 1) revealed the presence of residual grafted material, fibrous tissue, and bone, the latter being predominant. The grafted material was in direct contact with newly formed bone in some areas and no gaps were observed, whilst fibrous tissue was sometimes observed around it; in particular small graft fragments were often visible, mixed with bone and fibrous tissue. Well organized lamellae and various lacunae with osteocytes, typical of sound bone architecture, were found in newly formed bone. There was no inflammatory reaction, as either infiltrates or foreign body granuloma formation. Histomorphometry demonstrated that the samples were composed of 39,84% ± 2,96 bone tissue, 26,91% ± 3,26 fibrous tissue, and 33,24% ± 3,65 grafted material (Figure 2), indicating a significantly higher prevalence of bone in comparison with the other tissues. This result was further confirmed by normalized measurements, as the BV/TV was 59,73% ± 3,90 and FTV/TV was 40,27% ± 3,90, indicating a significantly higher bone tissue area (*p* < 0,05) (Figure 3). As for residual graft material, a reduction in particle size compared to what was stated by the manufacturer was observed (Figure 4), thus indicating a graft resorption pattern.

## 4. Discussion

The primary aim of this study was to investigate the histologic characteristics of grafted maxillary sinuses at 6 months, employing an equine-derived bone. Additionally, clinical parameters were analyzed.

Scientific literature regarding sinus augmentation in humans consists mainly in perspective studies or clinical reports on implant survival rate.

Histology of autogenous bone graft of maxillary sinus has consistently shown high amount of new bone formation. A systematic review and meta-analysis by Rickert et al. has shown that autogenous grafts are associated with an average bone fraction of 80% after sinus lift [20].

DBBM has been successfully employed in this type of procedures due to its limited resorption after years, even if the material has shown a poor bone formation potential: DBBM has shown a bone fraction of 40–42%, with chronic inflammation in the marginal bone zone [21–23]. Bioactive glasses combined with autologous bone have demonstrated a bone fraction of 35% at 4-5 months after sinus augmentation [24]. *β*-TCP has shown a bone fraction of 74% and the characteristic of woven bone [25]. Grow factors such as PRP or BMP-7 in association with autogenous bone seem to produce a superior outcome regarding bone fraction at 4 months after sinus floor surgery [26], but no clinical benefits could be observed in a meta-analysis by Rickert et al. in 2012 [20]. DFDB has been associated with histological signs of chronic inflammation and osteoconductive properties that became evident after one week of insertion [27]. Fresh-Frozen Bone data on sinus augmentation have demonstrated the presence of residual FFB particles and new bone formation with no areas of inflammation at 6 months [28].

To the best of our knowledge, only limited studies investigated equine-derived bone graft for bone augmentation procedures. A work by Schwarz et al. using an equine bone graft for ridge augmentation on beagle dogs reported signs of degradation by macrophages, osteoclasts, and multinucleated giant cells. Interestingly, the graft resorption rate in the equine bone specimen was higher than that in the bovine bone control group [29]. Moreover, EB scaffold showed a high bone formation activity.

Our histological data have shown a reduction in graft particle size compared to those stated by the manufacturer, thus indicating a marked resorption rate.

Inflammatory infiltrates, or signs of foreign body reaction, were not detected in any of the histological sections investigated in the present study. This could be significant in terms of safety of the graft material, proving that it could induce a moderate immunologically driven response.

The high percentage of regenerated bone (40%) can indicate that the graft material could be largely reabsorbed by 6 months as it could be replaced by newly formed vital bone. Contrariwise, scientific literature shows that DBBM may delay the healing process filling the space necessary for the new bone formation [30–32]. These results are important for the maintenance of osteointegration over time, as the main goal of dental implants is their long-term maintenance at the bone-graft interface [33].

## 5. Conclusion

This study showed that equine-derived bone can be considered an effective graft material in sinus augmentation technique, when considering the amount of bone. The graft was shown to be well-tolerated by patients and acted as a bioactive material that induces new bone formation.

Future studies are warranted to test the use of this graft material for other bone regenerative procedures, such as for the reconstruction of edentulous atrophic alveolar ridges, to obtain alveolar bone regeneration around teeth and implants and for socket preservation.
